# Are Patients Requiring Hartmann’s Procedure Being Adequately Optimised for Surgery: An Audit Cycle

**DOI:** 10.7759/cureus.41589

**Published:** 2023-07-09

**Authors:** Cameron A Lynch

**Affiliations:** 1 General Surgery, Tameside and Glossop Integrated Care National Health Service (NHS) Foundation Trust, Manchester, GBR

**Keywords:** asa score, pre operative evaluation, surgery general, hartmann procedure, quality improvement project, clincal audit

## Abstract

Introduction

Hartmann’s procedures are common surgical operations indicated in a wide variety of presentations including colon malignancy, diverticular disease, volvulus, and colovesical and colovaginal fistulas. The procedure is a major undertaking for the patient and those presenting in the emergency setting are often clinically unwell with deranged laboratory investigations. Numerous studies have demonstrated that pre-operative anaemia contributes to increased morbidity and mortality. Applying the conclusions of one study recommending a minimum haemoglobin >12 g/dL level pre-operatively, this audit assessed patient optimisation prior to Hartmann’s procedure.

Materials and methods

Patients undergoing Hartmann’s procedures between May 2016 and February 2020 were identified. Data was collected retrospectively to analyse American Society of Anesthesiology (ASA) grade and pre-operative haemoglobin level. Pre-operative haemoglobin and group and save blood test values were identified pre-and post-intervention.

Results

Pre-intervention, 15 (21%) of 70 patients had a haemoglobin level <12 g/dL and 63 patients (90%) had a group and save blood test completed on admission. Post-intervention data was collected from 45 patients, with figures improving to five (11%) and 44 (97%) patients, respectively.

Conclusion

Our flowchart poster distribution and addition to the surgical proforma led to increased patient optimisation prior to Hartmann’s procedure.

## Introduction

A Hartmann’s procedure is the surgical resection of the rectosigmoid colon with the closure of the anorectal stump and the formation of an end colostomy. It was first described in 1923 by Henri Albert Hartmann as surgical management for colorectal cancer, with an aim to reduce mortality by avoiding the formation of colorectal anastomosis [1,2]. Common indications for the procedure include but are not limited to colon malignancy, diverticular disease, volvulus, and colovesical and colovaginal fistulas [3,4]. The procedure involves removing the pathological lesion, and closing the distal bowel intraperitoneally, with the proximal bowel diverted to a stoma. Depending on the site of the lesion this results in an end colostomy or ileostomy.

A Hartmann’s procedure is a major operation whether emergency or elective, with significant implications for the patient, in addition to the prospect of potential future operations to re-join the bowel. One large retrospective study of nearly 4000 patients noted a reversal rate of 23.3% [5]. We decided to audit our trust’s local management of pre-operative optimisation for Hartmann’s procedures.

There are limited national guidelines regarding Hartmann’s procedures. A paper examining the outcomes of the reversal of Hartmann’s procedures noted factors including reducing patient’s weight, smoking cessation, and reducing steroid doses were beneficial optimisation strategies prior to surgery [6]. Similar influential factors were described in a paper investigating Hartmann’s procedure reversal and rate of stoma-free survival, citing smoking status and length of stay increased complication rates [7]. It could be argued factors increasing complication rates could minimise post-operative mortality if optimised before surgery.

We note the findings of Harries et al. who investigated ‘prognostic factors for survival following emergency Hartmann’s procedure’ [8]. Their findings were summarised into three points: emergency colorectal procedures are associated with significant morbidity and mortality, age over 75 years offers a significant survival disadvantage when performing emergency Hartmann’s procedure, and preoperative American Society of Anesthesiology (ASA) status of greater than or equal to three and haemoglobin <12 g/dl were more significant independent predictors of mortality than age.

Parallels to Harries et al. have been noted by the Enhanced Recovery After Surgery (ERAS®) Society recommendations, stating anaemia is a common laboratory finding in patients presenting for colorectal surgery [9]. Outside of the context of Hartmann’s procedures, a study examining non-cardiac surgery patients cited poor patient outcomes in those anaemic pre-operatively, findings correlating with a paper analysing over 3000 orthopaedic patients which showed that a low haemoglobin level prior to surgery increases mortality [10,11]. Depending on the exact pathophysiological mechanism for presentation to the hospital, the majority of patients presenting for colorectal surgery will be iron deficient [12].

Several papers have concluded that addressing anaemia via peri-operative blood transfusions is detrimental to patient outcomes [11,13,14]. Further emphasising the importance of pre-operative optimisation.

The focus of this audit is on the final point described by Harries et al., which indicates that a higher ASA grade and preoperative haemoglobin of <12 g/dL have negative implications on the mortality of patients undergoing Hartmann’s procedures. As ASA grade is difficult to improve in the acute setting, the focus of this audit will regard patient pre-operative optimisation with regards to completion of the initial group and save blood test with a view to optimise haemoglobin levels pre-operatively.

## Materials and methods

Patients at Tameside and Glossop Integrated Care Foundation Trust in the Northwest of England who had undergone a Hartmann’s procedure between May 2016 and February 2020 were identified. The local data collation department provided a list of patients who had undergone a Hartmann’s procedure during this timeframe. Using the Lorzeno electronic patient record (EPR) discharge summary subsection, patients’ age, sex, indication for Hartmann’s procedure, survival time, and comorbidities were collected. The investigation subsection of the EPR revealed the presence of a group and save blood test and the patient’s pre-operative full blood count which detailed the haemoglobin level. ASA grade was documented on the pre-operative anaesthetic assessment, taking into account variables including age and comorbidities. 

During the first cycle, data was analysed retrospectively from 70 patients who had undergone an elective or emergency Hartmann’s procedure. The inclusion criteria included all patients who had undergone a Hartmann’s procedure during the aforementioned time frame. There were no exclusion criteria. A descriptive analysis of the audit data was performed using Microsoft Excel. The parameters for each patient were inputted into a table to allow identification of ASA grade, haemoglobin level, and presence of a group and save blood test. 

Beginning in September 2020 an intervention in the form of a flowchart addition to the surgical proforma was introduced to prompt the clerking clinician to interpret the patient’s haemoglobin level and order a group and save blood test. The second cycle was conducted post-intervention between August 2021 and June 2023; patient information was collected retrospectively using the EPR. No patients were excluded during the second cycle. A total of 45 patients had undergone a Hartmann’s procedure either electively or in the emergency setting during this time frame.

## Results

Pre intervention

Seventy patients underwent Hartmann’s procedure from 16th May 2016 to the 13th February 2020. The median age was 66 years (range 32-88 years). Eleven patients had the operation as elective and 59 attended as emergencies. The modal group made up of 48 patients had a Hartmann’s procedure for perforation of sigmoid diverticular disease. Followed by 12 patients who had colorectal malignancies, five patients had sigmoid volvulus, two had colovesical or colovaginal fistula, and three had miscellaneous causes. On admission, patients were seen by the surgical team and resuscitated with intravenous fluids, and prescribed antibiotics and analgesia as appropriate. Prior to the Hartmann’s procedure, patients were reviewed by a consultant surgeon and anaesthetist to stratify risks.

Post-operatively the 30-day mortality rate was 7.14% (five patients), followed by 11.42% (eight patients) after six months, and 14.28% (10 patients) after 12 months. The survival rates after one and two years were 85.72% (60 patients) and 82.86% (58 patients), respectively. With regards to pre-operative blood levels, 20 patients had haemoglobin levels below the target range for their sex (male 13-18 g/dl, female 11.5-16.5 g/dl) as per local hospital policy. 21.42% (15 patients) had a haemoglobin level <12 g/dL. 90.00% (63 patients) had a group and save requested on admission.

**Figure 1:**
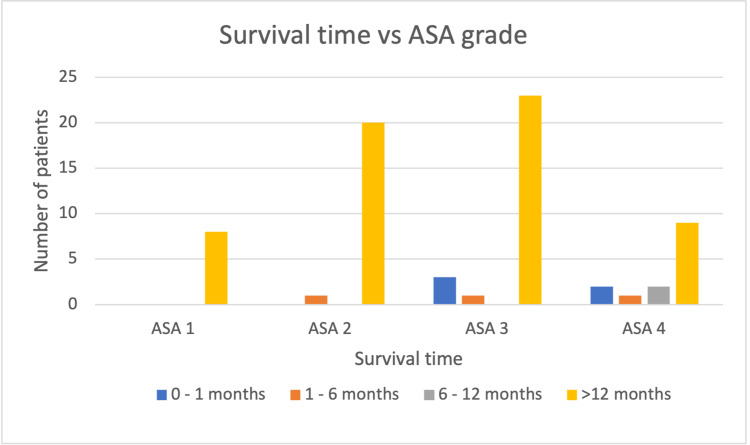
Bar graph demonstrating the number of patients versus mean survival time per ASA grade following Hartmann’s procedure pre-intervention. ASA: American Society of Anesthesiology

Stratifying the patients by ASA grade with regards to survival time, it can be observed that on average those with a lower ASA grade survived longer (Figure 1).

Intervention

**Figure 2:**
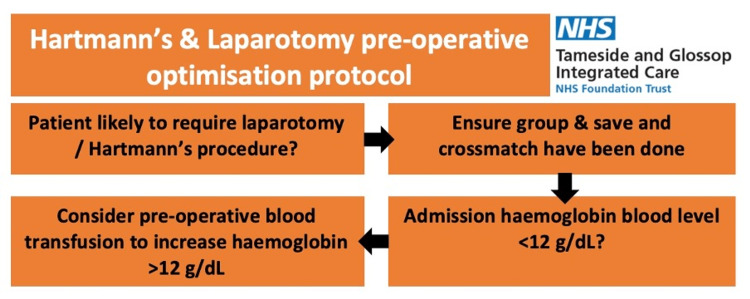
Flowchart included in the surgical proforma to prompt pre-operative patient optimisation.

The second audit cycle was conducted retrospectively after intervention in the form of a flowchart (Figure 2) in addition to the surgical clerking proforma. A flowchart poster was created to ensure group and save, and crossmatch were completed, with the aim of greater pre-operative patient optimisation. Figure 2 demonstrates the flowchart prompting clinicians to consider blood transfusion for laparotomy and Hartmann’s patients if feasible in the emergency setting. The flowchart was distributed via a global email to the surgical clinicians. Visual posters were introduced in the Surgical and Accident and Emergency departments, in addition to modifying the surgical clerking proforma. During induction for rotating junior doctors, the intervention was highlighted to ensure continuity of use.

Post intervention

Post-intervention data was collected retrospectively for patients presenting between 4th August 2021 and 7th June 2023. The median age was 61 years (34-86 years). Fourty-five patients had undergone Hartmann’s procedures during this time. Five patients had a Hartmann’s procedure completed in the elective setting and 40 had an emergency operation. The modal pathology, totalling 28 patients, resulting in a Hartmann’s procedure was perforation of sigmoid diverticular disease. Colorectal malignancy was the attributed pathology in six patients, followed by five patients with sigmoid volvulus. Three patients had colovesical or colovaginal fistula, and three had miscellaneous causes. Patients were admitted and clerked by a junior surgical doctor, all being prescribed intravenous antibiotics and fluids. Within a 24-hour period, all patients were reviewed by a consultant surgeon and anaesthetist prior to surgery. 

The post-operative 30-day mortality rate was 6.66% (three patients); at six months the rate was 11.11% (five patients), and after 12 months the figure was 15.55% (seven patients). After one and two years the survival rate was 86.66% (39 patients) and 82.22% (37 patients), respectively. Analysing pre-operative blood results, seven patients (15.55%) had haemoglobin levels below the target range for their sex (male 13-18 g/dl, female 11.5-16.5 g/dl) as per local hospital policy. Five patients (11.11%) had haemoglobin levels <12 g/dL. 97.77% (44 patients) had a group and save requested on admission.

**Figure 3:**
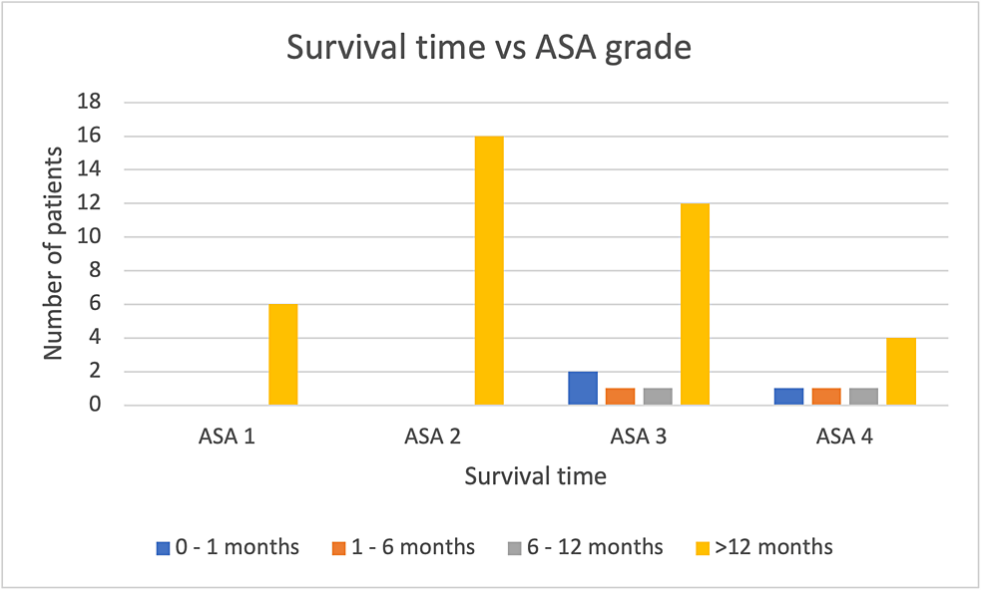
Bar graph demonstrating the number of patients versus mean survival time per ASA grade following Hartmann’s procedure post-intervention. ASA: American Society of Anesthesiology

Stratifying the re-audit group of patients by ASA grade, those with a lower ASA grade had increased mean survival time as demonstrated in Figure 3.

Table 1 demonstrates the improvements noted between cycles one and two with regards to patients’ haemoglobin levels prior to surgery, and completion of a group and save blood test upon admission.

**Table 1:** Patients' haemoglobin level comparison Percentage of patients with pre-operative haemoglobin levels >12 g/dL, and completion of a group and save blood test upon admission for cycles one and two.

Standard	Target	Cycle 1	Cycle 2
Preoperative haemoglobin level >12 g/dL	100%	78.57%	88.89%
Group and save requested on admission	100%	90.00%	97.77%

## Discussion

Hartmann’s procedures are common surgical operations indicated in a wide variety of pathologies. Patient optimisation is an important aim prior to any operation, enhancing the patient’s capability to undergo surgical intervention. It has been proven those with haemoglobin levels below the lower limit of the normal range have an increased risk of mortality in the post-operative period. This audit examined patient optimisation prior to surgery and successfully observed improvement post-intervention.

In this audit, we used a memory aid in the form of a flowchart inserted into the surgical clerking proforma to increase patient optimisation. Through repetition and in-person reminders at handover and junior doctor inductions, adherence to the flowchart increased. A clear improvement was observed for standards one and two. As expected, those patients with a higher ASA grade had reduced mean survival compared to their counterparts with fewer comorbidities. Due to the nature and chronic components of the ASA grade it is near impossible to decrease a patient’s grade acutely upon admission to hospital. This audit concurs with the findings of Harries et al. noting mean survival time decreasing with higher ASA grades [8]. 

Despite no patient being excluded in this audit, the sample is prone to selection bias as those presenting to the hospital with pathologies requiring a Hartmann’s procedure are likely individuals with a higher ASA grade due to predisposing comorbidities. However, this audit did not assess patients presenting to the surgical team who may have benefitted from a Hartmann’s procedure but were not deemed appropriate candidates for surgery. The standards measured in the study were based on one study alone which itself is a weakness. Other drawbacks include the nature of the retrospective study and that due to the study design, there was an impact of survival bias. It was presumed all patients' anaemia was corrected via intravenous blood transfusion; this may not have been the case. 

The post-operative survival rates at one month, six months, one year, and two years were higher during the second audit cycle. It is difficult to attribute this to optimisation prior to surgery as it will depend on a combination of factors including but not limited to ASA grade, post-operative mobilisation, post-operative infection, and complication rates. 

The strengths of the audit lay in the successful intervention which can strongly be attributed to the increased standard compliance detailed in Table 1. The limitations of this audit involved basing the methods on the findings of one paper; however, it can be argued no formal guidelines exist for optimisation prior to Hartmann’s procedure, in addition to multiple studies noting anaemia pre-operatively increases morbidity and mortality. The methods of haemoglobin replacement were not documented; however, due to the majority of patients falling into the emergency category intravenous infusion can be assumed. Patient optimisation and survival rates may have also been attributed to other parameters for example infection markers managed with antibiotics; however, this data was not collected. 

## Conclusions

The intervention of a flowchart introduced into the surgical clerking document in this audit cycle has led to increased patient optimisation prior to Hartmann’s procedure. However, it is difficult to directly associate this intervention with increased survival rates. Further research in a controlled environment is needed to determine the implication on mortality. We strongly recommend further implementation of clinical audits within the surgical department and wider National Health Services (NHS) trusts to continually assess and improve standards and patient care.

## References

[1] Sanderson ER (1980). Henri Hartmann and the Hartmann operation. Arch Surg.

[2] van de Wall BJ, Draaisma WA, Schouten ES, Broeders IA, Consten EC (2010). Conventional and laparoscopic reversal of the Hartmann procedure: a review of literature. J Gastrointest Surg.

[3] Barbieux J, Plumereau F, Hamy A (2016). Current indications for the Hartmann procedure. J Visc Surg.

[4] Khosraviani K, Campbell WJ, Parks TG, Irwin ST (2000). Hartmann procedure revisited. Eur J Surg.

[5] Horesh N, Rudnicki Y, Dreznik Y, Zbar AP, Gutman M, Zmora O, Rosin D (2018). Reversal of Hartmann's procedure: still a complicated operation. Tech Coloproctol.

[6] Suthakaran R, Faragher IG, Yeung JM (2023). Reversal of Hartmann's procedure: timelines, preoperative investigations and early outcomes. A single Australian institution's ten-year experience. ANZ J Surg.

[7] Hallam S, Mothe BS, Tirumulaju R (2018). Hartmann's procedure, reversal and rate of stoma-free survival. Ann R Coll Surg Engl.

[8] Harries RL, Twine CP, Kugathasan G, Young H, Jones E, Gomez KF (2012). Prognostic factors for survival following emergency Hartmann's procedure. Postgrad Med J.

[9] Gustafsson UO, Scott MJ, Hubner M (2019). Guidelines for Perioperative Care in Elective Colorectal Surgery: Enhanced Recovery After Surgery (ERAS®) Society recommendations: 2018. World J Surg.

[10] Baron DM, Hochrieser H, Posch M (2014). Preoperative anaemia is associated with poor clinical outcome in non-cardiac surgery patients. Br J Anaesth.

[11] Smilowitz NR, Oberweis BS, Nukala S (2016). Association between anemia, bleeding, and transfusion with long-term mortality following noncardiac surgery. Am J Med.

[12] Muñoz M, Gómez-Ramírez S, Martín-Montañez E, Auerbach M (2014). Perioperative anemia management in colorectal cancer patients: a pragmatic approach. World J Gastroenterol.

[13] Acheson AG, Brookes MJ, Spahn DR (2012). Effects of allogeneic red blood cell transfusions on clinical outcomes in patients undergoing colorectal cancer surgery: a systematic review and meta-analysis. Ann Surg.

[14] Bennett S, Baker LK, Martel G (2017). The impact of perioperative red blood cell transfusions in patients undergoing liver resection: a systematic review. HPB (Oxford).

